# Atorvastatin calcium tablets on inflammatory factors, hemorheology and renal function damage indexes in patients with diabetic nephropathy

**DOI:** 10.12669/pjms.37.5.4045

**Published:** 2021

**Authors:** Ronghua Li, Tianting Shi, Enpeng Xing, Hongcui Qu

**Affiliations:** 1 Ronghua Li Department of Nephrology, Binzhou People’s Hospital, Shandong, 256600, China; 2 Tianting Shi Department of General Surgery, Binzhou People’s Hospital, Shandong, 256600, China; 3 Enpeng Xing Department of Neurology, Binzhou People’s Hospital, Shandong, 256600, China; 4 Hongcui Qu Outpatient Operating Room, Maternity and Child Care Hospital in Zhangqiu, Shandong, China

**Keywords:** Atorvastatin, Diabetic nephropathy, Hemorheology, Renal function damage

## Abstract

**Objectives:**

To investigate the effect of atorvastatin on inflammatory factors, hemorheology, and renal function damage in patients with diabetic nephropathy (DN).

**Methods:**

One hundred and six DN patients who were treated in our hospital between June 2018 and August 2019 were selected and randomly grouped into observation group and control group, 53 each group. Patients in the control group were given the conventional treatment; patients in the observation group were given atorvastatin treatment on the basis of the conventional treatment. They were treated for three months. The hemorheology indexes (whole blood viscosity, erythrocyte aggregation index, and fibrinogen (FIB)), renal function damage indexes (macrophage migration inhibitory factor (MIF), vascular cell adhesion molecule (VCAM)-1, Secreted frizzled-related protein-5 (SFRP5), and mAIb/Cr) and inflammatory factor related indexes (C-reactive protein (CRP), interleukin-1 (IL-1), and tumor necrosis factor-α (TNF-α)) were compared between the two groups before and after three months of treatment.

**Results:**

After three months of treatment, the hemorheology indexes, renal function damage indexes, and inflammatory factors related indexes in the two groups changed. Compared with the control group, the whole blood viscosity, erythrocyte aggregation index, FIB, MIF, VACM-1, mAIb/Cr, CRP, IL-1, and TNF-α levels in the observation group significantly decreased, while the levels of SERP-5 significantly increased; the differences were statistically significant (P<0.05).

**Conclusion:**

Atorvastatin can effectively alleviate the renal function damage in patients with DN, reduce the level of serum inflammatory factors, and improve hemorheology, which has a good clinical application value for DN patients.

## INTRODUCTION

Diabetic nephropathy (DN) is a serious complication of diabetes mellitus. At present, the prevalence of diabetic nephropathy in China is increasing year by year.^1^,^2^ Studies have shown that the majority of patients with diabetic history of five to ten years had DN, which was mainly due to the serious damage of renal blood vessels and nephron, and with the prolongation of the course of diabetes mellitus, renal interstitium may be involved, thus leading to DN.^3^-^5^ DN is one of the factors leading to the end-stage renal disease. Once it progresses to the end-stage renal disease, the treatment difficulty increases, the risk of death increases correspondingly, and the quality of life of patients decreases. Some studies have pointed out that the correction of metabolic disorders is one of the key problems in the treatment of DN, thus, it is very important to properly handle dyslipidemia for the control of the occurrence and progression of DN.^6^,^7^ Statins are commonly used drugs to regulate blood lipid in clinic, which can improve blood lipid disorder, indirectly protect the kidney, and reduce oxidative stress, inflammatory reaction, and advanced glycation end products.^8^,^9^ A study finds that atorvastatin has a positive effect on hemodynamics in patients with early renal damage aroused by hypertension,^10^ suggesting that atorvastatin can be used as one of the drugs for the treatment of DN. In view of this, this study selected 106 cases of DN patients in our hospital as the research subjects and observed the clinical value of atorvastatin in the treatment of DN on the basis of the conventional treatment.

## METHODS

One hundred and six patients with DN who were treated in our hospital between June 2018 and August 2019 were selected. They all conformed to the diagnostic criteria of DN and were in DN stage III.^11^ They were randomly divided into an observation group and a control group, 53 cases each group. In the control group, there were 29 males and 24 females, aged 46-68 (57.03±6.32) years; the course of disease was 4-11 years, with an average of (6.97±2.56) years; 30 cases were in Mogenson stage III, and 23 cases were in Mogenson stage IV. The observation group was treated with atorvastatin in addition to the conventional treatment, and there were 28 males and 25 females; the age of the patients was 45-70 (57.67±6.21) years; the course of disease was 4-12 years, with an average of (7.05±2.48) years; 33 cases were in Mogenson stage III, and 20 cases were in Mogenson stage IV.

### Inclusion criteria

(1) 24-h urinary albumin between 30 mg and 300 mg; 2) cooperating to finish the study.

### Exclusion criteria

Those having urinary system diseases such as nephritis and hypertensive nephropathy; those having adverse reactions to the drugs used in this study; pregnant and lactating women and those taking lipid-lowering drugs and antioxidant drugs in the past one month.

This study was approved by the medical ethics committee (Ref: 217, Dated: 1-11-2020) of the hospital, and the patients and their families were informed with the content of the study and agreed the study. The results were comparable because there was no significant difference in general data between the two groups (P>0.05).

Both groups received the conventional treatment, including blood glucose control, blood pressure regulation, diet management, exercise management, etc. In addition to the conventional treatment, the observation group took atorvastatin calcium tablets (Beijing Jialin Pharmaceutical Co., Ltd., State Food and Drug Administration approval number H20093819) at a dosage of 20 mg/d. Both groups were treated for three months.

The hemorheology indexes (whole blood viscosity, erythrocyte aggregation index, aggregation index, and fibrinogen (FIB)) and renal function damage indexes (macrophage migration inhibitory factor (MIF), vascular cell adhesion molecule (VCAM)-1, Secreted frizzled-related protein 5 (SFRP5), and mAIb/Cr) of the two groups before and after three months of treatment were detected and compared. The levels of hemorheology indexes was detected using a MVIS-2015 automatic hemorheology analyzer. The levels of serum MIF, VCAM-1, and SFRP5 were detected using enzyme-linked immunosorbent assay (ELISA). The level of mAIb was detected by immunoturbidimetry. The level of Cr was detected by the enzymic method. The levels of inflammatory factors related indicators were detected by collecting the fasting venous blood samples before and after treatment from the two groups. The level of C-reactive protein (CRP) was measured by the immunofluorescence assay, and the levels of interleukin-1 (IL-1), and tumor necrosis factor-α (TNF-α) were detected by ELISA.

### Statistical analysis

SPSS 20.0 statistical software was used to process the data of this study. The measurement data in accordance with the normal distribution was represented by. The comparison between the two groups was conducted by t-test, the count data was expressed by rate (%), and the data comparison was conducted by x2 test. The difference was statistically significant (P<0.05).

## RESULTS

After three months of treatment, the whole blood viscosity, erythrocyte aggregation index, and FIB level of the two groups improved compared with those before treatment, and the whole blood viscosity, erythrocyte aggregation index, and FIB level of the observation group after treatment were significantly lower than those of the control group. The above differences were all statistically significant (P<0.05, Table-I).

**Table-I:** Comparison of hemorheology indexes between the two groups before and after treatment.

Group		Observation group	Control group	t	P
Whole blood viscosity (mPa·s)	Before treatment	1.86±0.13	1.87±0.15	0.084	0.921
	After treatment	1.36±0.34^#^	1.57±0.67^#^	4.917	<0.001
Erythrocyte aggregation index	Before treatment	1.81±0.67	1.79±0.50	0.323	0.756
	After treatment	1.48±1.03^#^	1.61±0.52^#^	8.172	<0.001
FIB (g/L)	Before treatment	0.62±0.19	0.61±0.17	0.314	0.752
	After treatment	0.48±0.16^#^	0.57± 0.14^#^	7.157	<0.001

Note:

# compared with before treatment, P<0.05.

After three months of treatment, the renal function damage index levels of the two groups improved compared with those before treatment; the levels of MIF, VACM-1, and mAIb/Cr in the observation group were significantly lower than those in the control group, and the level of SERP-5 was significantly higher than that in the control group. The above differences were statistically significant (P<0.05, Table-II).

**Table-II:** Comparison of renal function damage indexes between the 2 groups before and after treatment.

Group		Observation group	Control group	t	P
MIF (μg/L)	Before treatment	31.73±5.08	32.02±4.86	0.951	0.341
	After treatment	10.36±1.85^^#^^	19.26±3.45^^#^^	9.176	<0.001
VCAM-1 (μg/L)	Before treatment	69.93±7.32	68.02±7.64	0.418	0.689
	After treatment	25.65±4.85^#^	39.88±5.39^^#^^	14.454	<0.001
mAIb/cr (μg/mL)	Before treatment	46.47±11.26	45.97±11.06	0.192	0.859
	After treatment	30.16±5.21^^#^^	37.23± 8.63^^#^^	7.100	<0.001
SFRP5 (μg/L)	Before treatment	4.03±0.52	3.97±0.51	0.908	0.360
	After treatment	5.23±0.74^^#^^	4.51±0.53^^#^^	5.067	<0.001

Note:

# compared with before treatment, P<0.05.

After three months of treatment, the levels of inflammatory factors related indexes in the two groups improved compared with those before treatment, and the levels of CRP, IL-6, and TNF-α in the observation group after treatment were significantly lower than those in the control group. The above differences were statistically significant (P<0.05, Table-III).

**Table-III:** Comparison of inflammatory factors related indexes between the two groups before and after treatment.

Group		Observation group	Control group	t	P
CRP (mg/L)	Before treatment	8.46±0.63	8.58±0.69	0.217	0.830
	After treatment	4.75± 0.46^#^	6.22± 0.41^#^	2.109	0.031
IL-6 (pg/mL)	Before treatment	25.48 ±2.36	25.62±2.17	0.201	0.839
	After treatment	17.21± 2.06^#^	19.33± 2.32^#^	9.102	<0.001
TNF-α (pg/mL)	Before treatment	62.35±5.94	62.47±6.13	0.403	0.689
	After treatment	42.37 ±4.67^#^	55.14 ±4.38^#^	5.914	<0.001

Note:

# compared with before treatment, P<0.05.

## DISCUSSION

DN is a common clinical complication of diabetes mellitus, and its pathogenesis is complex. The occurrence of DN is closely related to hemodynamic abnormalities, oxidative stress response, abnormal cytokine secretion, and excessive accumulation of advanced glycation end products.^12^,^13^

DN patients had mass production of hyperglycemia, glycosylated hemoglobin (HbA1c), and poor red blood cell liquid fluidity, which will not only lead to the rise of red blood cell viscosity, but also cause high expression of FIB to induce the increase of blood viscosity.^14^ The rise of whole blood viscosity can directly lead to the increase of blood flow resistance and the slowing down of blood circulation rate, resulting in blood stasis and eventually forming microthrombosis; as a result, local tissue ischemia and hypoxia happens, which further aggravates the abnormal hemorheology indexes, induces malignant microcirculation disorder, and exacerbates renal injury.^15^,^16^ Chen et al. has found that atorvastatin adjuvant therapy is beneficial to the improvement of platelet activity and coagulation abnormalities in patients with diabetes mellitus complicated with coronary heart disease.^17^ Therefore, exploring the effect of atorvastatin on hemorheology related indexes of DN patients is helpful to evaluate the clinical efficacy. The results showed that the whole blood viscosity, erythrocyte aggregation index, and FIB level in the observation group were significantly lower than those in the control group after three months of treatment (P<0.05), indicating that atorvastatin could effectively improve the hemorheology of DN patients, and it might be related to the lipid-lowering effect of atorvastat. The decrease of blood lipid cannot only inhibit the decrease of glomerular filtration rate and reduce proteinuria, but also improve blood composition to improve hemorheology.

The results showed that the levels of MIF, VACM-1 and mAIb/Cr in the observation group were significantly lower than those in the control group (P<0.05), and the level of SERP-5 in the observation group was significantly higher than that in the control group (P<0.05), suggesting that atorvastatin could relieve the renal function damage and renal fibrosis of DN patients better than the conventional treatment. The reason for the above phenomena was that atorvastatin could protect the kidney by regulating blood lipid metabolism, inhibiting mesangial cell proliferation, inducing cell apoptosis, promoting extracellular matrix degradation, and moreover it could also play the renal protection mechanism by inhibiting vasoconstriction factors activity to improve endothelial cell function, regulate the blood flow in the kidney, and improve the renal microenvironment, which is consistent with the previous research theory.^18^

Studies have pointed out that inflammatory factors play an important role in the occurrence and development of DN, and the levels of inflammatory factors related indexes gradually increased with the aggravation of DN.^19^ DN patients are in a state of micro inflammation, which has a great impact on coagulation and fibrinolysis system and can promote the proliferation of cellulose-like substances and lead to renal function damage. A study has found that atorvastatin can reduce the expression of Toll Like Receptor-4 (TLR-4) protein in diabetic patients, and thus reduce the production of inflammatory mediators mediated by the activation of TLR-4 pathway. Some scholars used statins to treat patients with atherosclerosis, and the plaque of patients significantly reduced after treatment,^20^ which was related to inflammation relief effect of the drug.^21^,^22^ These findings suggest that statins can relieve inflammation, which is consistent with the conclusion of this study. The data of this study showed that the levels of CRP, IL-6, and TNF-α in the observation group after treatment were significantly lower than those before treatment, indicating that atorvastatin could help to control the inflammatory reaction of DN patients. In practice, the author finds that atorvastatin can inhibit the adhesion and aggregation of monocytes, block the production path of CRP, and down regulate the levels of serum IL-6 and TNF-α, which are related to the anti-inflammatory function of atorvastatin.

### Limitations of the study

It includes the small sample size and the short observation time. The sample size will be expanded in the future to analyze the long-term effects of atorvastatin at different doses on DN.

## CONCLUSION

To sum up, atorvastatin has an obvious clinical effect in the treatment of DN, which can help reduce the level of inflammatory factors and effectively improve hemorheology, thus to inhibit renal cell apoptosis and reduce renal injury; therefore, it has high clinical application values.

### Authors’ Contribution:

**RHL & TTS:** Study design, data collection, analysis and are responsible for integrity of study.

**EPX & HCQ:** Manuscript preparation, drafting and revising.

**RHL & HCQ:** Review and final approval of manuscript.
